# Is gastric mapping needed in the endoscopy of dyspeptic patients? 

**Published:** 2021

**Authors:** Seyed Mohammad Valizadeh Toosi, Seyede Fateme Sanayifar, Reza Ali Mohammadpour, Somayeh Sheidaei

**Affiliations:** 1 *Gut and Liver Research Center, Mazandaran University of Medical Sciences, Sari, Iran *; 2 *Mazandaran University of Medical Sciences, School of Medicine, Sari, Iran *; 3 *Department of Biostatistics, Mazandaran University of Medical Sciences, School of Health, Sari, Iran *; 4 *Department of Pathology, Mazandaran University of Medical Sciences, School of Medicine, Sari, Iran*

**Keywords:** Dyspepsia, Gastric mapping, Intestinal metaplasia

## Abstract

**Aim:**

Our study aimed to determine the prevalence of intestinal metaplasia in dyspeptic patients who underwent upper GI endoscopy.

**Background:**

Intestinal metaplasia, which is defined as the replacement of normal gastric mucosa by metaplastic intestinal epithelium, has been described as a premalignant gastric lesion.

**Methods:**

Six hundred two consecutive patients with dyspeptic symptoms who had undergone upper GI endoscopy were included in the study. For all patients, gastric mapping was performed to determine the presence of intestinal metaplasia. All histologic samples were reported according to the updated Sydney classification.

**Results:**

Total of 61.3% of the patients were female. The mean age of the patients was 46±15 years. The overall prevalence of intestinal metaplasia was 22%. The distribution of intestinal metaplasia in the stomach was 15.1% in the antrum, 4.3% in the body, and 2.6% in the antrum and body together. Also, the prevalence of intestinal metaplasia in the age group of under 40 years was 9.5% and in patients over 40 years it was 29.5%..

**Conclusion:**

The results of this study have shown that more than one-fifth of the patients with dyspepsia have intestinal metaplasia. This indicates that gastric mapping in patients with dyspepsia may lead to the detection of precancerous lesions especially after the age of 40.

## Introduction

 Dyspepsia is a feeling of discomfort in the epigastric region, mainly as a pain and or burning sensation, nausea and vomiting, or a feeling of epigastric fullness. Dyspepsia has several causes including functional dyspepsia, Gastric ulcer, duodenal ulcer, and Gastric or esophageal cancer.

Gastric cancer is the fourth most common cancer and the third leading cause of cancer deaths worldwide (1). In Iran, gastric cancer is the most common cancer in men and the second most common cancer in women after breast cancer (2). According to the multistage model of gastric adenocarcinoma development (Correa cascade), a combination of genetic factors, chronic Helicobacter pylori (H pylori) infection, nutritional factors (high salt intake, alcohol, and smoking), chronic bile reflux, and environmental factors are involved in the development of inflammatory pan-gastritis (3).

The Correa model of intestinal-type gastric carcinogenesis is a multistep cascade in which chronic active gastritis progresses to multifocal Atrophic Gastritis, intestinal metaplasia (IM), low-grade dysplasia, high-grade dysplasia, and finally gastric adenocarcinoma (4, 5). The global world prevalence of H pylori infection is more than 50%. In Iran, the prevalence of H.pylori infection varies from 13% up to 82% (6). *H.pylori* is the first bacterial carcinogen that has been described (7). 

It is the cause of more than 80% of duodenal ulcers and more than 60% of gastric ulcers and almost all primary malt lymphoma (8). Seventy-seven percent of gastric cancer of both histologic types, in the distal part of the stomach, are due to chronic *H.pylori* infection (9). The mean age of patients with H.pylori-positive IM is less than those with *H.pylori*-negative IM (10). 

Gastric IM, which is defined as the replacement of normal gastric mucosa by metaplastic intestinal epithelium, has been described as a premalignant lesion (3). Gastric intestinal metaplasia is a pathological finding; therefore, to evaluate the presence of this tissue change, Gastric mapping is required during upper GI endoscopy.

 The prevalence of gastric IM varies across different regions of the world. In the study of Craanen M et al., in 1992, the prevalence of IM in Amsterdam was 25.3% (10) , and in the study of Edit et al. (2015), the prevalence of IM in the antrum was higher than in the body (22.9% vs. 2.8%) (11). Also, as revealed by Patterson et al. in Sweden, in 2002, the prevalence of IM in the general population was 23% (12). Malekzadeh and his colleagues in Ardabil, in the north west of Iran (area of high prevalence for gastric cancer), found that the prevalence of H pylori infection was 89.2% and the prevalence of atrophic gastritis was 39.3% in the antrum and 21.9% in the cardia. In this study, the prevalence of intestinal metaplasia was 8.7% in the antrum 3.8% in the cardia (13). 

In a systematic review by Peleterio, in 2007, which included the results of studies from 29 countries, the prevalence of IM varied from 3% to 24.4% in different regions (14). Our study aimed to evaluate the prevalence of intestinal metaplasia in patients with dyspepsia who are candidates for upper GI endoscopy. 

## Methods

In this cross-sectional study, patients with dyspepsia, who had indicated for upper gastrointestinal endoscopy, enrolled in the study. According to the Iranian gastrointestinal association guideline, dyspeptic patients over 40 years or those who have alarming symptoms require upper GI endoscopy. From June to November 2018, based on the inclusion and exclusion criteria, among 751 patients who referred to gastroenterology clinics of Mazandaran University of Medical Sciences, 602 patients enrolled in the study. The inclusion criteria were comprised of the following as applied to 14-80-year-old patients: dyspepsia and alarming symptoms such as nausea /vomiting, melena, weight loss, anemia, and age above 40 years). The exclusion criteria included a contraindication for upper GI endoscopy, history of previous HP eradication, and antibiotic usage in last month or proton-pump inhibitor (PPI) usage in the last two weeks. Endoscopy was performed by Pentax Endoscope version (EG2985). In patients with abnormal endoscopic findings, a pathological specimen was obtained from the pathologic lesions. Endoscopic gastric mapping (two biopsies from the antrum, one from incisure angularis, and two biopsies from the body) was performed for all patients. Samples that were obtained from the gastric antrum and angularis were kept in one container and samples from the gastric body in another one. Then, both containers were sent to the pathology ward.

All pathologic samples were reported by our pathologist coworker based on the updated Sydney Classification for histologic changes of chronic gastritis, atrophic gastritis, and intestinal metaplasia (15, 16). Also, pathological specimens were evaluated by Giemsa staining method for H pylori infection.

A questionnaire was completed for each patient including demographic information of age, sex, history of smoking, aspirin and NSAID usage, prominent symptoms of dyspepsia including epigastric pain, epigastric burning sensation and fullness, Nausea & vomiting and endoscopic findings and H.pylori infection data. 

 After data collection, the data were analyzed by SPSS16 software. The central and dispersion indices were used to describe the data: the Chi-Square test for the quantitative and t-tests for qualitative data, respectively. P-values <0.05 were considered statistically significant.

The scientific committee of the Gut and Liver Research Center and the ethics committee of Mazandaran University of Medical Sciences approved the proposal of this study with the ethics code IR.MAZUMS.IMAMHOSPITAL.REC.1397.1805.

## Results

Six hundred two patients with dyspepsia were enrolled in the study. The mean age of patients was 46±15 years. Two hundred thirty-three patients were male (38.7%) and 369 patients were female (61.3%). Epigastric pain was the most common symptom (67.6%), with other symptoms of epigastric-burning sensation, fullness, nausea, and vomiting with lesser frequency. In our study, the history of smoking, NSAID, and aspirin usage was 13.3%, 29.1%, and 19.3% respectively. The overall prevalence of *H.pylori* infection was 68.8%. (Table 1), 69.1% in females and 68.2% in males. The overall prevalence of intestinal metaplasia was 22%. (Table 1).

**Table 1:** Epidemiologic, endoscopic and pathologic findings (n=62)

Sex	
Male Female	233 (38.7%) 369 (61.3%)
Age (mean±SD)	46±15 yrs (range: 14- 80)
Smoking	80/602 (13.3%)
NSAIDs	175/602 (29.1%)
Aspirin	116/602 (19.3%)
Indication for EGD	
Epigastric pain Epigastric burning sensation Epigastric fullness Nausea & vomiting Others	407 (67.6%) 72 (12%) 24 (4%) 54 (9%) 45 (7.4%)
endoscopic findings	
GU Gastritis (erosive, non-erosive, and atrophic etc.) DU & duodenitis Normal EGD	82 (13.6%) 211 (35%) 48 (8%) 261 (43.4%)
*H. pylori* infection	
Positive negative	414 (68.8%) 188 (31.2%)
Intestinal metaplasia	
Positive Negative	133 (22%) 469 (78%)

**Figure 1:**
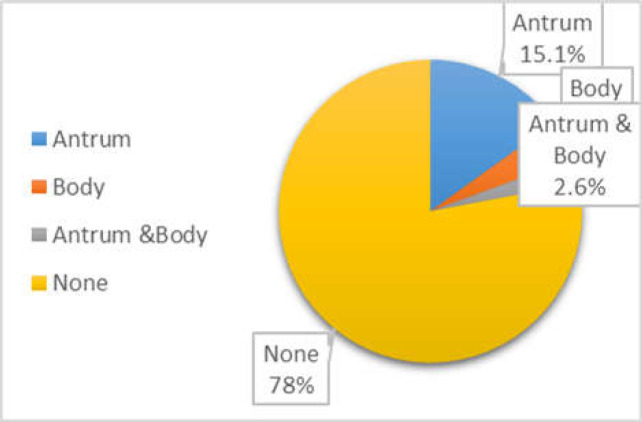
Prevalence of IM in different regions of the stomach

 The prevalence of IM was 18.6% in females and 26.1% in males (P-value < .0.01) The distribution of IM in the stomach in the antrum, body, and antrum and body together was 15.1%, 4.3%, and 2.6% respectively. The most common site of intestinal metaplasia in the stomach was the antrum. (Figure 1). 

The prevalence of IM in patients with normal upper GI endoscopy was 16.5% and in patients with gastric ulcer 36.5%, duodenal ulcer and erosion 18.7%, and erosive gastritis 23.2%. (Table 2). The prevalence of IM in the age group of under 40 years was 9.5% and in patients over 40 years it was 29.5% (P value < 0.001) (Table 3). The highest rate of intestinal metaplasia was detected in patients over 60 years (40%). Sixty-eight percent of patients with IM were HP positive. The prevalence of IM in HP-positive patients were 22.1% and in HP-negative patients it was 20.8%.

**Table 2:** Endoscopic findings and prevalence of IM

Decade of age	No. of IM positive (%)	Total No. of patients (%)
14 - 20	1 (2.9%)	34 (5.6%)
21 - 30	4 (6.2%)	65 (10.8%)
31 - 40	17 (12.7%)	134 (22.3%)
41 - 50	25 (20.3%)	123(20.4%)
51 - 60	34 (28.1%)	121 (20.1%)
> 60	50 (40.0%)	125 (20.8%)
Under 40 yrs.	22 (9.5%)	233 (38.7%)
Over 40 yrs.	109 (29.5%)	369 (61.3%)

**Table 3:** Prevalence of intestinal metaplasia (IM) in different decades of

Decade of age	No. of IM positive (%)	Total No. of patients (%)
14 - 20	1 (2.9%)	34 (5.6%)
21 - 30	4 (6.2%)	65 (10.8%)
31 - 40	17 (12.7%)	134 (22.3%)
41 - 50	25 (20.3%)	123(20.4%)
51 - 60	34 (28.1%)	121 (20.1%)
> 60	50 (40.0%)	125 (20.8%)
Under 40 yrs.	22 (9.5%)	233 (38.7%)
Over 40 yrs.	109 (29.5%)	369 (61.3%)

## Discussion

The prevalence of gastric IM in the general population varies in the world. This is mostly due to differences in the prevalence of H. Pylori infection (17). IM is a well-known risk factor for the development of gastric cancer (18). Due to the high prevalence of gastric cancer and H. pylori infection in our region, we decided to evaluate the prevalence of IM.

The prevalence of gastric IM ranges from 12.5% up to 25.3% in different studies. The prevalence of IM in the study of Craanen M was 25.3% (10), Peterson et al. 23% (12), Peleterio’s review study 15.6% (14), Ericsson et al. 19% (19), Ozdil et al. 17.8% (20), Ajdarkosh H et al. 19.8% (21), Olmez et al. 13.8% (22), and Malekzadeh et al. 12.5% (13). The studies mentioned above are all retrospective without gastric mapping. Only Ericsson, Malekzadeh, and Ozdil followed a prospective design and performed gastric mapping for all patients. 

As we know, chronic H.pylori infection leads to intestinal metaplasia over many years (5, 23) . These chronic inflammatory changes can occur in any part of the stomach. Therefore, in histological evaluation of gastric mucosa; all parts of gastric mucosa should be examined. Gastric mapping provides an opportunity to detect any pathologic changes in all parts of the gastric mucosa (24). 

In our study, the overall prevalence of gastric IM was 22% (Table 1). IM was higher in males than females, but not with a statistically significant difference. Similar to previous studies, in our study, the prevalence of intestinal metaplasia increases with age (10-12). The prevalence of intestinal metaplasia was 9.5% in patients under 40 years and 29.5% in patients over 40 years (P-value <0.001). The highest prevalence of IM was seen after the age of 60 years (40%). This finding could underscore that endoscopy and gastric mapping in patients with dyspepsia may lead to the detection of gastric precancerous lesions in early stages.

The most common endoscopic finding accompanied by intestinal metaplasia was gastric ulcer (36.5%), and the second most common was erosive gastritis (23.2%) in our study, which is compatible with Edit et al. study (11).

In this study, the most common location of intestinal metaplasia in the stomach was the antrum (Figure 1), i.e., the gastric antrum was the site of involvement in 79% of patients with intestinal metaplasia. This finding is consistent with the location of gastric cancer the most common site of which is the antrum (18).

The prevalence of HP infection in this study was 68.8%, but its prevalence in previous studies varied from 54.2% in the study of Craanen M up to 89.2% in the study of Malekzadeh et al. (10, 13, 20, 21, 25), possible due to the socio-economic status of patients, general health conditions of the area, and the method of H.pylori infection evaluation.

One of the findings of this study was that intestinal metaplasia was present in 16.5% of patients with normal endoscopy, which has not been mentioned in previous studies. It emphasizes the importance of gastric mapping in dyspepsia patients with normal upper GI endoscopy.

Similar to previous studies, in this study, the prevalence of IM in patients with a history of cigarette smoking and NSAID usage was not significantly different from those without these histories. Contrary to our expectation, patients with a history of aspirin usage had a higher prevalence of IM than those who had not used aspirin (30% vs. 20%). 

Conclusion:

 The results of this study show that more than one-fifth of patients with dyspepsia have IM and up to a third have IM after the age of 40 years. These indicate that gastric mapping in patients with dyspepsia can lead to the detection of precancerous lesions, especially in the fourth decade of life.

## References

[1] Zayeri F, Sheidaei A, Rahimzadeh S, Rezaei N, Modirian M, Baghestani AR (2016). Evaluation of the trends of stomach cancer incidence in districts of Iran from 2000-2010: Application of a random effects Markov Model. Asian Pac J Cancer Prev.

[2] Mousavi SK, Janbabai G, Kouchaki B, Borhani H, Rashidi M, Salehifar E (2015). Demographic and clinical characteristics of gastric cancer patients in north of Iran, Mazandaran province, 2008-2014. Pharm Biomed Res.

[3] Correa P (2004). The biological model of gastric carcinogenesis. IARC Sci Publ.

[4] Toh JW, Wilson RB (2020). Pathways of Gastric Carcinogenesis, Helicobacter pylori Virulence and Interactions with Antioxidant Systems, Vitamin C and Phytochemicals. Int J Mol Sci.

[5] Correa P (1992). Human gastric carcinogenesis: a multistep and multifactorial process—first American Cancer Society award lecture on cancer epidemiology and prevention. Cancer Res.

[6] Moosazadeh M, Lankarani KB, Afshari M (2016). Meta-analysis of the prevalence of Helicobacter pylori infection among children and adults of Iran. Int J Prev Med.

[7] Compare D, Rocco A, Nardone G (2010). Risk factors in gastric cancer. Eur Rev Med Pharmacol Sci.

[8] Shafii M, Nikzad SE, Kasiri H, Naghipour M (2008). Histopathological evaluation of chronic gastritis with and without Helicobacter pylori colonization: a study from Iran. Malays J Pathol.

[9] Correa P, Piazuelo MB (2013). The gastric cancer. Colomb Medica.

[10] Craanen M, Dekker W, Blok P, Ferwerda J, Tytgat G (1992). Intestinal metaplasia and Helicobacter pylori: an endoscopic bioptic study of the gastric antrum. Gut.

[11] Eidt S, Stolte M (1994). Prevalence of intestinal metaplasia in Helicobacter pylori gastritis. Scand J Gastroenterol.

[12] Petersson F, Borch K, Franzen L (2002). Prevalence of subtypes of intestinal metaplasia in the general population and in patients with autoimmune chronic atrophic gastritis. Scand J Gastroenterol.

[13] Malekzadeh R, Sotoudeh M, Derakhshan M, Mikaeli J, Yazdanbod A, Merat S (2004). Prevalence of gastric precancerous lesions in Ardabil, a high incidence province for gastric adenocarcinoma in the northwest of Iran. J Clin Pathol.

[14] Peleteiro B, Bastos J, Barros H, Lunet N (2008). Systematic review of the prevalence of gastric intestinal metaplasia and its area-level association with smoking. Gac Sanit.

[15] Correa P, Piazuelo MB, Wilson KT (2010). Pathology of gastric intestinal metaplasia: clinical implications. Am J Gastroenterol.

[16] Stolte M, Meining A (2001). The updated Sydney system: classification and grading of gastritis as the basis of diagnosis and treatment. Can J Gastroenterol Hepatol.

[17] Ohkuma K, Okada M, Murayama H, Seo M, Maeda K, Kanda M (2000). Association of Helicobacter pylori infection with atrophic gastritis and intestinal metaplasia. J Gastroenterol Hepatol.

[18] Mabula JB, Mchembe MD, Koy M, Chalya PL, Massaga F, Rambau PF (2012). Gastric cancer at a university teaching hospital in northwestern Tanzania: a retrospective review of 232 cases. World J Surg Oncol.

[19] Eriksson N, Kärkkäinen PA, Färkkilä M, Arkkila PE (2008). Prevalence and distribution of gastric intestinal metaplasia and its subtypes. Dig Liver Dis.

[20] Ozdil K, Sahin A, Kahraman R, Yuzbasıoglu B, Demirdag H, Calhan T (2010). Current prevalence of intestinal metaplasia and Helicobacter pylori infection in dyspeptic adult patients from Turkey. Hepato-gastroenterol.

[21] Ajdarkosh H, Sohrabi M, Moradniani M, Rakhshani N, Sotodeh M, Hemmasi G (2015). Prevalence of gastric precancerous lesions among chronic dyspeptic patients and related common risk factors. Eur J Cancer Prev.

[22] Olmez S, Aslan M, Erten R, Sayar S, Bayram I (2015). The prevalence of gastric intestinal metaplasia and distribution of Helicobacter pylori infection, atrophy, dysplasia, and cancer in its subtypes. Gastroent Res Pract.

[23] Fuccio L, Zagari R, Minardi M, Bazzoli F (2007). Systematic review: Helicobacter pylori eradication for the prevention of gastric cancer. Aliment Pharma Ther.

[24] Buxbaum JL, Hormozdi D, Dinis-Ribeiro M, Lane C, Dias-Silva D, Sahakian A (2017). Narrow-band imaging versus white light versus mapping biopsy for gastric intestinal metaplasia: a prospective blinded trial. Gastrointest Endosc.

[25] Bozorgnia MA, Kashfi SMH, Ariana M, Ghalkhani F, Iravani S, Lashkari MH (2015). Prevalence and correlation of chronic atrophic gastritis, intestinal metaplasia and other precancerous lesions of stomach in Iran: a historical cohort study. Clin Transl Gastroenterol.

